# Synthesis, Anticancer Activity, and Apoptosis Induction of Novel 3,6-Diazaphenothiazines †

**DOI:** 10.3390/molecules24020267

**Published:** 2019-01-12

**Authors:** Beata Morak-Młodawska, Krystian Pluta, Małgorzata Latocha, Małgorzata Jeleń, Dariusz Kuśmierz

**Affiliations:** 1 Department of Organic Chemistry, School of Pharmacy with the Division of Laboratory Medicine, The Medical University of Silesia, Jagiellońska 4, 41-200 Sosnowiec, Poland; pluta@sum.edu.pl (K.P.); manowak@sum.edu.pl (M.J.); 2 Department of Cell Biology, School of Pharmacy with the Division of Laboratory Medicine, The Medical University of Silesia, Jedności 8, 41-200 Sosnowiec, Poland; mLatocha@sum.edu.pl (M.L.); dkusmierz@sum.edu.pl (D.K.)

**Keywords:** phenothiazines, dipyridothiazines, antiproliferative activity, dialkylaminoalkynyl substituents, apoptosis, H3, TP53, CDKN1A, BAX/BCL-2 ratio

## Abstract

New 10-substituted derivatives of 3,6-diazaphenothiazine, containing the triple bond linker terminated with tertiary cyclic and acyclic amine groups, were synthesized and screened for their anticancer action. The compounds exhibited varied anticancer activities against human glioblastoma SNB-19, melanoma C-32, and breast cancer MDA-MB231 cell lines, depending on the nature of the substituents. The most active 3,6-diazaphenothiazine, **4**, was the derivative with the *N*,*N*-diethylamino-2-butynyl substituent against glioblastoma SNB-19, and was ten times more potent than cisplatin. For this compound, the expression of *H3, TP53, CDKN1A, BCL-2,* and *BAX* genes was detected by the RT-qPCR method. The gene expression ratio *BAX/BCL-2* indicated the induction of mitochondrial apoptosis in cancer cell lines. The transformation of the propynyl substituent into amino-2-butynyl can be a method applicable to the search for more anticancer-active azaphenothiazines.

## 1. Introduction

Cancer has become a global problem, ranked among the top leading causes of death worldwide, right after cardiovascular disease, and killing more people than AIDS, tuberculosis, and malaria combined. It is expected to surpass heart diseases as the leading cause of death in the next few years, due to the growth and aging of the population. Treatments include surgery, radiation, chemotherapy, hormone therapy, immune therapy, and targeted therapy (drugs that interfere with cancer cell growth by targeting specific molecules) [1,2,3]. Chemotherapy, in particular, has improved significantly, and the survival rates have increased greatly, but there is still need to discover and develop new more potent antitumor agents with better selectivity and reduced side effects [4].

Molecules possessing nitrogen and sulfur atoms (for example, sulfonamides and phenothiazines) are among of the most biologically active and are significant scaffolds in medicinal chemistry. The last compounds are not only well known antipsychotic drugs, but exhibit various other valuable pharmaceutical properties. Thioridazine, one of the best known phenothiazines, exhibits promising properties for multidrug-resistant tuberculosis treatment [5] and lung cancer therapy through targeting lung cancer stem cells, while being both efficient and safe [6].

The chemical modifications of the phenothiazine system were mainly based on introduction of new substituents at the thiazine nitrogen atom and on replacement of one or two benzene rings with the aza-aromatic rings forming azaphenothiazines [7]. These modifications are aimed at finding new biological activities. Both classical and modified phenothiazines have been described recently in several reviews as compounds possessing a promising range of properties, such as anticancer, antibacterial, antifungal, anti-inflammatory, antioxidant activities, and the reversal of multidrug resistance [7,8,9,10,11,12,13,14,15,16,17]. They are regarded as potentially beneficial in treatment of Alzheimer’s, Creutzfeldt–Jakob, and AIDS-associated diseases [18,19,20].

The replacement of the benzene ring with the pyridine ring leads to pyridobenzothiazines and dipyridothiazines. Some pyridobenzothiazines are known as antipsychotic (prothipendyl), antihistaminic (isothipendyl), antiemetic (pervetral), and antitussive (pipazethate) drugs [17]. Recently, they were found to exhibit antiviral activity against chikungunya virus (CHIKV), a mosquito-transmitting alphavirus causing CHIK fever, as well as a promising anticancer activity [21,22].

Dipyridothiazines were found to demonstrate an excellent scaffold for novel anticancer agents with improved safety profile. We synthesized dipyridothiazines of the 1,6-, 1,8-, and 2,7-diazaphenothiazine structures with varied alkyl, aryl, heteroaryl, dialkylaminoalkyl, amidoalkyl, sulfonamidoalkyl, and “half-mustard” substituents at the thiazine nitrogen atoms. Some of those compounds exhibit very promising properties. In addition to their anticancer activity, they also possess immunosuppressant and antioxidant properties, and low toxicity [23,24,25,26,27,28,29].

Mannich bases, which are obtained through the introduction of an aminomethyl functional group by means of the Mannich reaction to heterogeneous class of substrates, exhibit a variety of biological activities, such as anticancer, antibacterial, antifungal, antiviral, anticonvulsant, anti-inflammatory, analgesic, and antioxidant [30]. Most of the classical and modified bioactive phenothiazines contain flexible pharmacophoric dialkylaminoalkyl substituents at the thiazine nitrogen atom. The Mannich bases possessing the dialkylamino-2-butynyl structure, and being a more rigid substituent, were very seldom explored in the phenothiazine area. There are only two papers on the synthesis and dialkylaminobutynylphenothiazines and dialkylaminobutynyldipyridothiazines (1,8- and 2,7-diazaphenothiazines). Some of those compounds exhibit the MDR-reverting activity, antitumor profile, and low lipophilicity [31,32].

Recently, we synthesized a new group of 3,6-diazaphenothiazine derivatives possessing anticancer activity. The parent molecule, 10*H*-3,6-diazaphenothiazine, exhibited high activity against human glioblastoma SNB-19, melanoma C-32, breast MCF-7, and ovarian A2780 cancer cells with the IC_50_ values less than 0.72 µg/mL. The molecule induced G2/M phase cell cycle arrest and caspase-dependent apoptosis, and inhibited cell invasion of ovarian carcinoma cell through regulation of NF-κB and [BIRC6-XIAP] complex [33,34]. The 3,6-diazaphenothiazine derivatives with flexible substituents at the thiazine nitrogen atom (alkyl and dialkylaminoalkyl) were found to possess less activity against tested cancer lines in comparison with 10*H*-3,6-diazaphenothiazine [33].

In this paper, novel 3,6-diazaphenothiazines with more rigid substituents containing a triple bond were synthesized and tested for their anticancer action on the selected cancer cell lines, in order to find more active compounds than the parent molecule. To understand the mechanism of action and the effects on cancer biology, for the most active compound, the expression of *H3*, *TP53*, *CDKN1A*, *BCL-2*, and *BAX* genes was detected by the RT-qPCR method.

## 2. Results and Discussion

### 2.1. Chemistry

**Scheme 1:**
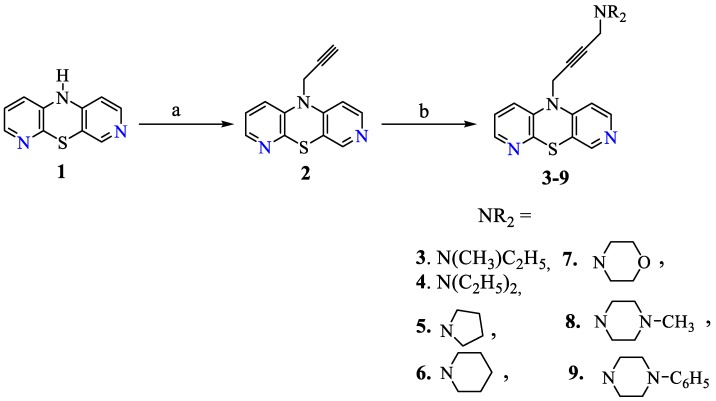
Synthesis of 3,6-diazaphenothiazines with dialkylaminoalkynyl substituents.

The synthetic strategy to obtain the target compounds is depicted in Scheme 1. The starting material, 10*H*-3,6-diazaphenothiazine **1**, was transformed with propynyl bromide into the 2-propynyl derivative **2**, according to the described synthesis [33], and further using Mannich condensation (with formaldehyde and selected secondary acyclic and cyclic amines, in the presence of CuCl as a catalyst) into the 4-dialkylamino-2-butynyl derivatives of 3,6-diazaphenothiazines (**3**–**9**) in good yields (68–84%). The structures of the new compounds were characterized with the use of spectroscopic data: ^1^H-NMR, ^13^C-NMR, FAB-MS, and HR-MS.

### 2.2. Anticancer Activity

The activity of target compounds (**3**–**9**) was investigated in vitro using cultured human glioblastoma SNB-19, melanoma C-32, and breast cancer MDA-MB231 cell lines and cisplatin as a reference. The tested compounds exhibited varied degrees of activity depending on the type of tested cell lines and the nature of the substituent at the thiazine nitrogen atom (Table 1).

The derivatives **3** and **4**, with the *N*-methyl-*N*-ethylamino-2-butynyl and *N*,*N*-diethylamino-2-butynyl groups, were found to be the most active against the SNB-19 cell line (IC_50_ = 0.11 μg/mL). Strong activity was observed also for derivative **4** against the C-32 cell line. In those cases, the activity was even higher than for cisplatin. Significant activity (IC_50_ = 0.45–0.74 μg/mL) was also demonstrated by the derivative **9** with the *N*-phenylpiperazinyl-2-butynyl fragments against cell lines 1 and 3. The compounds **5**–**7** with the aminobutynyl substituents, containing the pyrrolidine, piperidine, and morpholine moieties, showed very weak activity in the range of tested concentrations. Derivative **2** (possessing only the propynyl group), being the starting material in the synthesis of the tested compounds, showed low anticancer activity (IC_50_ = 32.9–45.1 μg/mL), as described earlier [33]. This means that the transformation of the propynyl substituent into amino-2-butynyl can increase the anticancer activity. The finally obtained derivatives were compared with the isomeric derivatives of the 1,6-, 1,8- and 2,7-diazaphenothiazines previously described. Compound **4** is the most potent derivative in the group of dipyridothiazines described up to now. The activity is at least 300 times more potent than its isomers [28,32]. Based on the conducted research, it can be concluded that the places of additional nitrogen atoms in the phenothiazine moiety have an influence on antiproliferative activity.

### 2.3. Apoptosis Assay

The most active derivative **4** was selected for studies on the mechanism of antitumor activity through the use of gene expression analysis: proliferation marker (*H3*), cell cycle regulators (*TP53, CDKN1A*), and markers of the apoptosis pathway (*BCL-2* and *BAX*). Antiproliferative activity of the tested compound for C-32 cells was confirmed in the analysis of the expression of a gene encoding the histone H3, which is an accepted marker of proliferation in molecular studies. The product of *TP53* gene is the P53 protein, which acts as a genome guardian and is also involved in the regulation of many cell processes. One of this protein’s major tasks is stopping the cell cycle when DNA damage occurs that is not repaired, and the P53 protein can initiate apoptosis. In cells, apoptosis can be initiated by intrinsic mitochondrial pathway with *BAX* and *BCL-2* involved (pro- and antiapoptotic proteins family). A new target in anticancer therapies is to restore the P53 activity in tumor cells, which could lead to the precise degradation of cancer cells. This protein product also regulates the P21 protein gene (*CDKN1A*) expression. This protein selectively binds to cyclin-dependent kinase complexes with cyclins and regulates the cell cycle. This means that an inadequate number of the P21 protein, or its mutations in cells, can induce an oncogenic transformation [35,36,37,38].

The results of the analysis of *H3, TP53, CDKN1A, BCL-2,* and *BAX* genes in SNB-19, C-32, MDA-MB231 cells after 24 h of treatment are collected in Table 2. Compound **4** reduced, significantly, the number of mRNA copies of *H3* in all cell lines, suggesting an alteration in chromatin structure. There is also a reduction in the number of copies of the gene *TP53*, significantly in the C-32 cells, but slightly in others. An increase in the number of *CDKN1A* copies in the SNB-19 and MDA-MB231 cells points to the possibility of the induction of cell cycle arrest and apoptosis. 

The P53 protein can also stimulate the cell to changes in the gene expression of proapoptopic *BAX* and antiapoptopic *BCL-2* involved in mitochondrial pathway apoptosis. *BAX* protein’s role in cells is the increase of the mitochondrial membrane permeability by the formation of pores, while *BCL-2* protein is responsible for the release of cytochrome C into the cytosol [39]. As a result of the described study, an increased *BAX/BCL-2* ratio indicates the activation of mitochondrial apoptosis in the SNB-19 cells. Transcriptional activity of these genes in MDA-MB231 and C-32 cells suggests a different way of cell death and, possibility, of a protective activation.

## 3. Materials and Methods

### 3.1. Chemistry

The ^1^H-, ^13^C-NMR spectra were recorded on a Bruker Fourier 300 and Bruker DRX spectrometers (Bruker, Billerica, MA, USA) at 600 MHz in deuterochloroform with tetramethylsilane as the internal standard. Fast Atom Bombardment mass spectra (FAB MS, in glycerol) were run on a Finnigan MAT 95 spectrometer (Thermo Finnigan LLC, San Jose, CA) at 70 eV and HR MS were run on a Brucker Impact II (Bruker, Daltonix Inc., Billerica, MA, USA). Thin layer chromatography was performed on aluminum oxide 60 F_254_ neutral (type E) (Merck 1.05581, (Merck, Darmstad, Germany) with CHCl_3_–EtOH (10:1 *v*/*v*) as eluent.

10*H*-3,6-diazaphenothiazine (**1**) and 10-propynyl-3,6-diazaphenothiazines (**2**) were obtained according to the reported procedures in [33].

#### General Procedure for Synthesis of Compounds (**3**–**9**)

A mixture of 10-propynyl-diazaphenothiazine **2** (0.5 mmol), paraformaldehyde (0.5 mmol), amine (0.7 mmol), and cuprous chloride (catalytic amount) in peroxide-free, dry dioxane (10 mL) was heated with continuous stirring at 70 °C for 3 h. After cooling, 20 mL water was added and mixture was extracted with chloroform, dried with Na_2_SO_4_, and evaporated in vacuo. The dry residue was dissolved in CHCl_3_ and purified by column chromatography (aluminum oxide, CHCl_3_) to give:

a. *10-[4-(N-ethyl-N-methyl)amino-but-2-ynyl]-3,6-diazaphenothiazine* (**3**): (0.110 g, 71%); an oil. ^1^H-NMR (CDCl_3_) δ: 1.05 (t, *J* = 7.2 Hz, 3H, CH_3_), 2.27 (s, 3H, N-CH_3_), 2.43 (q, *J* = 7.2 Hz, 2H, N-CH_2_), 3.38 (s, 2H, N-CH_2_), 4.43 (s, 2H, N-CH_2_), 6.93 (d, *J* = 5.4 Hz, 1H, H_1_), 7.07 (dd, *J* = 7.8, *J* = 4.9 Hz, 1H, H_9_), 7.33 (dd, *J* = 7.8 Hz, *J* = 4.8 Hz, 1H, H_8_), 8.07 (dd, *J* = 4.8 Hz, *J* = 1.2 Hz, 1H, H_7_), 8.10 (s, 1H, H_4_), 8.29 (d, *J* = 5.4 Hz, 1H, H_2_). ^13^C-NMR (150 MHz, CDCl_3_) δ: 12.2, 37.9, 41.5, 45.2, 49.9, 78.9, 82.5, 109.2, 118.7, 121.1, 122.1, 137.8, 143.9, 145.9, 146.9, 148.8, 149.2. FAB-MS *m/z*: 311 (M + 1, 100), 253 (M − C_3_H_5_N, 20). HRMS (EI) *m/z* for [C_17_H_18_N_4_S + H] calcd 311.1330. Found: 311.1298.

b. *10-[4-(N,N-diethyl)amino-but-2-ynyl]-3,6-diazaphenothiazine* (**4**): (0.129 g, 80%); an oil. ^1^H-NMR (CDCl_3_) δ: 1.08 (t, *J* = 7.2 Hz, 6H, 2CH_3_), 2.54 (q, *J* = 7.2 Hz, 4H, 2N-CH_2_), 3.50 (s, 2H, N-CH_2_), 4.41 (s, 2H, N-CH_2_), 6.93 (d, *J* = 5.4 Hz, 1H, H_1_), 7.09 (dd, *J* = 7.8, *J* = 4.9 Hz, 1H, H_9_), 7.33 (dd, *J* = 7.8 Hz, *J* = 4.8 Hz, 1H, H_8_), 8.09 (dd, *J* = 4.8 Hz, *J* = 1.2 Hz, 1H, H_7_), 8.19 (s, 1H, H_4_), 8.29 (d, *J* = 5.4 Hz, 1H, H_2_). ^13^C-NMR (CDCl_3_) δ: 12.8, 41.6, 45.4, 49.9, 77.9, 82.6, 109.1, 118.7, 120.9, 122.1, 137.9, 143.9, 145.9, 146.9, 148.9, 149.2. FAB-MS *m/z*: 325 (M + 1, 100), 252 (M − C_4_H_10_N, 10). HR-MS (EI) *m/z* for [C_18_H_20_N_4_S + H] calcd 325.1487. Found: 325.1457. (Supplementary Materials).

c. *10-(4-pyrrolidin-1-yl-but-2-ynyl)-3,6-diazaphenothiazine* (**5**): (0.120 g, 74%); an oil. ^1^H-NMR (CDCl_3_) δ: 1.79 (m, 4H, 2CH_2_), 2.59 (m, 4H, 2CH_2_), 3.45 (s, 2H, CH_2_), 4.44 (s, 2H, CH_2_), 6.94 (d, *J* = 5.4 Hz, 1H, H_1_), 7.08 (dd, *J* = 7.8, *J* = 4.9 Hz, 1H, H_9_), 7.34 (dd, *J* = 7.8 Hz, *J* = 4.8 Hz, 1H, H_8_), 8.08 (dd, *J* = 4.8 Hz, *J* = 1.2 Hz, 1H, H_7_), 8.19 (s, 1H, H_4_), 8.29 (d, *J* = 5.4 Hz, 1H, H_2_). ^13^C-NMR (CDCl_3_) δ: 22.8, 41.6, 45.4, 55.9, 78.9, 82.7, 109.1, 118.9, 120.8, 122.3, 137.9, 143.8, 145.9, 146.9, 148.9, 149.2. FAB-MS *m/z*: 323 (M + 1, 30), 200 (M − C_8_H_12_N, 100). HR-MS (EI) *m/z* for [C_18_H_18_N_4_S + H] calcd 323.1330. Found: 323.1325.

d. *10-(4-piperidin-1-yl-but-2-ynyl)-3,6-diazaphenothiazine* (**6**): (0.128 g, 76%); an oil. ^1^H-NMR (CDCl_3_) δ: 1.43 (m, 2H, CH_2_), 1.61 (m, 4H, 2CH_2_), 2.46 (m, 4H, 2CH_2_), 3.32 (s, 2H, CH_2_), 4.43 (s, 2H, CH_2_), 6.96 (d, *J* = 5.4 Hz, 1H, H_1_), 7.09 (dd, *J* = 7.8, *J* = 4.9 Hz, 1H, H_9_), 7.35 (dd, *J* = 7.8 Hz, *J* = 4.8 Hz, 1H, H_8_), 8.10 (dd, *J* = 4.8 Hz, *J* = 1.2 Hz, 1H, H_7_), 8.12 (s, 1H, H_4_), 8.30 (d, *J* = 5.4 Hz, 1H, H_2_). ^13^C-NMR (CDCl_3_) δ: 23.8, 25.9, 41.8, 45.7, 55.8, 78.9, 82.7, 108.9, 118.8, 120.8, 122.3, 137.9, 143.8, 145.9, 146.9, 148.9, 149.7. FAB-MS *m/z*: 337 (M + 1, 30), 201 (M + 1 − C_9_H_14_N, 100). HR-MS (EI) *m/z* for [C_19_H_20_N_4_S + H] calcd 337.1487. Found: 337.1488.

i. *10-(4-morpholin-4-yl-but-2-ynyl)-3,6-diazaphenothiazine* (**7**): (0.115 g, 68%); an oil. ^1^H-NMR (CDCl_3_) δ: 2.54 (m, 4H, 2CH_2_), 3.29 (s, 2H, CH_2_), 3.61 (m, 4H, 2O-CH_2_), 4.79 (s, 2H, CH_2_), 6.81 (dd, *J* = 7.5 Hz, *J* = 4.8 Hz, 1H, H_3_), 6.95 (d, *J* = 4.9 Hz, 1H, H_6_), 7.23 (dd, *J* = 7.5 Hz, *J* = 1.5 Hz, 1H, H_4_), 8.06 (dd, *J* = 5.1 Hz, *J* = 1.5 Hz, 1H, H_2_), 8.10 (d, *J* = 4.9 Hz, 1H, H_7_), 8.36 (s, 1H, H_9_). ^13^C-NMR (CDCl_3_) δ: 41.3, 45.1, 55.8, 66.1, 79.1, 82.2, 108.9, 118.8, 120.8, 122.3, 137.9, 143.8, 145.9, 146.9, 148.7, 149.9. FAB-MS *m/z*: 339 (M + 1, 35), 200 (M − C_8_H_12_NO, 100). HR-MS (EI) *m/z* for [C_18_H_18_N_4_SO + H] calcd 339.1280. Found: 339.1277.

k. *10-[4-(4-methylpiperazin-1-yl)but-2-ynyl]-3,6-diazaphenothiazine* (**8**): (0.124 g, 70%); an oil. ^1^H-NMR (CDCl_3_) δ: 2.30 (s, 3H, N-CH_3_), 2.55 (m, 8H, 4CH_2_), 3.35 (s, 2H, CH_2_), 4.42 (s, 2H, CH_2_), 6.92 (d, *J* = 5.4 Hz, 1H, H_1_), 7.07 (dd, *J* = 7.8, *J* = 4.9 Hz, 1H, H_9_), 7.30 (dd, *J* = 7.8 Hz, *J* = 4.8 Hz, 1H, H_8_), 8.07 (dd, *J* = 4.8 Hz, *J* = 1.2 Hz, 1H, H_7_), 8.16 (s, 1H, H_4_), 8.27 (d, *J* = 5.4 Hz, 1H, H_2_). ^13^C-NMR (CDCl_3_) δ: 41.6, 44.8, 46.7, 53.8, 56.7, 79.1, 82.2, 108.9, 118.8, 120.8, 122.3, 137.9, 143.8, 145.9, 146.9, 148.7, 150.1. FAB-MS *m/z*: 352 (M + 1, 100), 202 (M + 1 − C_9_H_15_N_2_, 35). HR-MS (EI) *m/z* for [C_19_H_21_N_5_S + H] calcd 352.1596. Found: 352.1596.

m. *10-[4-(4-phenylpiperazin-1-yl)but-2-ynyl]-3,6-diazaphenothiazine* (**9**): (0.173 g, 84%); an oil. ^1^H-NMR (CDCl_3_) δ: 2.72 (m, 4H, 2CH_2_), 3.07 (s, 2H, CH_2_), 3.23 (m, 4H, 2CH_2_), 4.43 (s, 2H, CH_2_), 6.86 (m, 2H, H1, H_9_), 7.10 (m, 4H, H_Ph_), 7.11 (m, 1H, H_Ph_), 7.30 (dd, *J* = 7.8 Hz, *J* = 4.8 Hz, 1H, H_8_), 8.12 (dd, *J* = 4.8 Hz, *J* = 1.2 Hz, 1H, H_7_), 8.21 (s, 1H, H_4_), 8.32 (d, *J* = 5.4 Hz, 1H, H_2_). ^13^C-NMR (CDCl_3_) δ: 41.4, 44.8, 53.9, 56.7, 79.1, 82.2, 108.9, 114.3, 118.8, 120.8, 121.8, 122.3, 129.7, 137.9, 143.8, 145.9, 146.9, 148.7, 149.6, 150.1. FAB-MS *m/z*: 414 (M + 1, 100), 201 (M + 1 − C_13_H_17_N_2_, 20). HR-MS (EI) *m/z* for [C_24_H_23_N_5_S + H] calcd 414.1752. Found: 414.1751.

### 3.2. Anticancer Effects In Vitro

#### 3.2.1. Cell Culture

3,6-Diazaphenothiazines **3**–**9** were evaluated for their anticancer activity using three cultured cell lines: SNB-19 (human glioblastoma, DSMZ—German Collection of Microorganisms and Cell Cultures, Braunschweig, Germany), C-32 (human amelanotic melanoma, ATCC—American Type Culture Collection, Manassas, VA, USA), and MDA-MB231 (human breast adenocarcinoma, ATCC, Manassas, VA, USA). The cultured cells were kept at 37 °C and 5% CO_2_. The cells were seeded (1 × 10^4^ cells/well/100 µL DMEM supplemented with 10% FCS and streptomycin and penicillin) using 96-well plates (Corning). The cells were counted in a hemocytometer (Bürker chamber) using a phase contrast Olympus IX50 microscope (Olympus Optical CO. GMBH, Hamburg, Germany) equipped with Sony SSC-DC58 AP camera and Olympus DP10 digital camera (Olympus Optical CO. GMBH, Hamburg, Germany).

#### 3.2.2. Cell Proliferation and Viability

Antiproliferative effect of the obtained compounds on cancer cells was determined using the Cell Proliferation Reagent WST-1 assay (Roche Diagnostics, Mannheim, Germany). This assay is based on the viable cell’s ability to cleave the bright red-colored tetrazolium salt [2-(4-iodophenyl)-3-(4-nitrophenyl)-5-(2,4-disulfophenyl)-2H-tetrazolium, monosodium salt] to a dark red soluble formazan by cellular enzymes. An increase in the amount of formazan dye formed correlates to the number of metabolically active cells in the culture. The formazan dye produced by metabolically active cells is quantified by a scanning ELISA reader by measuring the absorbance of the dye solution at appropriate wavelengths. The examined cells were exposed to the tested compounds for 72 h at various concentrations between 0.1 μg/mL and 100 μg/mL (prepared initially at a concentration of 1 mg/mL in DMSO). The cells were incubated with WST-1 (10 μL) for 1 h and the absorbance of the samples against a background control was measured at 450 nm using a microplate reader with a reference wavelength of 600 nm. The results are expressed as means of at least two independent experiments performed in triplicate. The antiproliferative activity of the tested compound was compared to cisplatin. The IC_50_ values (concentration of the compound that is required for 50% inhibition) were calculated from the dose–response relationship with respect to control.

#### 3.2.3. RT-qPCR Method

Gene transcriptional activity (*H3, TP53, CDKN1A, BCL-2, BAX*) was evaluated by real time RT-qPCR method with OPTICON TM DNA Engine (MJ Research, Watertown, NY, USA) and QuantTect^®^ SYBR^®^ Green RT-PCR Kit (Qiagen, Valencia, spain). Cells were exposed to compound **4** at 0.5 µg/mL concentration for 24 h. The RNA extraction was made by using Quick-RNA™ Kit MiniPrep (ZYMO RESEARCH, Irvine, CA, USA). Total RNA integrity was analyzed using 1.2% agarose electrophoresis with added ethidium bromide. The quantity and purity of extracted total RNA were determined by using spectrophotometric analysis with HP845 (Hewlett Packard, Waldbronn, Germany) spectrophotometer. The statistical analysis was performed using the Statistica 8.0 software (StatSoft, Tulsa, OK, USA). All values were expressed as means ± SE.

## 4. Conclusions

We report here synthesis of new 10-substituted derivatives of 3,6-diazaphenothiazine with more rigid substituents than dialkylaminoalkyl, containing the triple bond linker ended with tertiary cyclic and acyclic amine groups. These compounds exhibited varied anticancer activities against human glioblastoma SNB-19, melanoma C-32, and breast cancer MDA-MB231 cancer lines, depending on the nature of the substituents. Three compounds (**3**, **4**, and **9**) were more active than the parent molecule, 10*H*-3,6-diazaphenothiazine. The most active compound was 3,6-diazaphenothiazine **4** with the *N*,*N*-diethylamino-2-butynyl substituent against the glioblastoma SNB-19, ten times more potent than cisplatin. This compound was more potent than its 1,6-, 1,8-, and 2,7-analogs. The location of the azine nitrogen atoms in the dipyridothiazine system seems to be crucial for anticancer activity. For this compound, the expressions of *H3, TP53, CDKN1A, BCL-2*, and *BAX* genes were detected by the RT-qPCR method. The gene expression ratio of BAX/BCL-2 indicated the induction of mitochondrial apoptosis in the SNB-19 cells. The transformation of the propynyl substituent into dialkylamino-2-butynyl via the Mannich reaction can be a method for searching for more anticancer active azaphenothiazines. 

## Figures and Tables

**Table 1:** Anticancer activities IC_50_ (µg/mL) of 10-substituted 3,6-diazaphenothiazines (**3**–**9**) against glioblastoma SNB-19, melanoma C-32, and ductal carcinoma MDA-MB231 cells.

No	Anticancer Activity IC_50_ (μg/mL)		
	SNB-19	C-32	MDA-MB231
**2**	45.1 [23]	32.9 [23]	37.9 ^a^ [23]
**3**	0.11 ± 0.1	1.53 ± 0.6	2.81 ± 0.8
**4**	0.11 ± 0.1	0.54 ± 0.2	6.32 ± 1.2
**5**	>50	>50	>50
**6**	>50	>50	>50
**7**	>50	>50	>50
**8**	2.43 ± 1.2	>50	7.64 ± 2.2
**9**	0.45 ± 0.1	4.57 ± 1.1	0.74 ± 0.3
***Cisplatin***	1.12 ± 0.4	3.96 ± 0.2	7.75 ± 1.6

^a^ human breast cancer MCF-7.

**Table 2:** The influence of compound **4** on the expression of genes encoding H3, TP53, CDKN1A, BCL-2, and BAX in glioblastoma SNB-19, melanoma C-32, and ductal carcinoma MDA-MB231 cells.

Gene	Compound	SNB-19	C-32	MDA-MB231
		Number of mRNA Copies/μg Total RNA		
*H3*	control	17909 ± 1812	995904 ± 181187	183104 ± 32057
	**4**	9006 ± 825	364206 ± 45530	48424 ± 6183
*TP53*	control	534284 ± 53484	682740 ± 84733	181536 ± 4213
	**4**	522817 ± 81864	349227 ± 33057	170163 ± 13984
*CDKN1A*	control	242104 ± 131105	1752117 ± 374944	33965 ± 2504
	**4**	324905 ± 10763	918317 ± 25533	49764 ± 2445
*BCL-2*	control	15316 ± 1085	68431 ± 12162	121242 ± 13880
	**4**	9392 ± 2312	32278 ± 1323	109429 ± 10990
*BAX*	control	918016 ± 47035	2431793 ± 574892	154192 ± 11428
	**4**	1061056 ± 165795	1053715 ± 155279	143037 ± 18827
*BAX/BCL-2*	control	55.9	35.5	1.27
	**4**	113	32.6	1.31

## Supplementary Materials

The supplementary materials are available online.

## References

[1] Torre L., Siegel R., Jemal A. (2015). Global Cancer Facts and Figures.

[2] Siegel R.L., Miller K.D., Jemal A. (2015). Cancer statistics, 2015. CA Cancer, J. Clin..

[3] Ferlay J., Soerjomataram I., Dikshit R., Eser S., Mathers C., Rebelo M., Parkin M.D., Forman D., Bray F. (2015). Cancer incidence and mortality worldwide: Sources, methods and major patterns in GLOBOCAN 2012. Int. J. Cancer.

[4] Bray F., Ren J.-S., Masuyer E., Ferlay J. (2013). Global estimates of cancer prevalence for 27 sites in the adult population in 2008. Int. J. Cancer.

[5] Amaral L., Viveiros M. (2017). Thioridazine: A non-antibiotic drug highly effective, in combination with first line anti-tuberculosis drugs, against any form of antibiotic resistance of mycobacterium tuberculosis due to its multi-mechanisms of action. Antibiotics.

[6] Yue H., Huang D., Qin L., Zheng Z., Hua L., Wang G., Huang J., Huang H. (2016). Targeting lung cancer stem cells with antipsychological drug thioridazine. Bio. Med. Res. Int..

[7] Pluta K., Morak-Młodawska B., Jeleń M. (2009). Synthesis and properties of diaza-, triaza- and tetraazaphenothiazines. J. Heterocycl. Chem..

[8] Motohashi N., Kawase M., Satoh K., Sakagami H. (2006). Cytotoxic potential of phenothiazines. Curr. Drug Targets.

[9] Mitchell S.C. (2006). Phenothiazine: The parent molecule. Curr. Drug Targets.

[10] Mosnaim A.D., Ranade V.V., Wolf M.E. (2006). Phenothiazine molecule provides the basic chemical structure for various classes of pharmacotherapeutic agents. Am. J. Therapeut..

[11] Dasgupta A., Dastridara S.G., Shirataki Y., Motohashi N. (2008). Antibacterial activity of artificial phenothiazines and isoflavones from plants. Bioact. Heterocycles.

[12] Aaron J.J., Gaye Seye M.D., Trajkovska S., Motohashi N. (2009). Bioactive phenothiazines and benzo[a]phenothiazines: Spectroscopic studies and biological and biomedical properties and applications. Bioact. Heterocycles.

[13] Sadandam Y.S., Shetty M.M., Bhaskar Rao A. (2009). 10H-Phenothiazines: A new class of enzyme inhibitorsfor inflammatory diseases. Eur. J. Med. Chem..

[14] Sudeshna G., Parimal K. (2010). Multiple non-psychiatric effect of phenothiazines: A review. Eur. J. Pharmacol..

[15] Pluta K., Morak-Młodawska B., Jeleń M. (2011). Recent progress in biological activities of synthesized phenothiazines. Eur. J. Med. Chem..

[16] Jaszczyszyn A., Gąsiorowski K., Świątek P., Malinka W., Cieślik-Boczula K., Petrus J., Czarnik-Matusewicz B. (2012). Chemical structure of phenothiazines and their biological activity. Pharmacol. Rep..

[17] Pluta K., Jeleń M., Morak-Młodawska B., Zimecki M., Artym J., Kocięba M., Zaczyńska E. (2017). Azaphenothiazines a promising phenothiazine derivatives. An insight into nomenclature, synthesis, structure elucidation and biological properties. Eur. J. Med. Chem..

[18] Viveiros M., Martins M., Couto I., Kristiansen J.E., Molnar J., Amaral L. (2005). The In vitro activity of phenothiazines against mycobacterium avium: Potential of thioridazine for therapy of the co-infected AIDS patient. In Vivo.

[19] González-Muñoz G.C., Arce M.P., López B., Pérez C., Romero A., del Barrio L., Martín-de-Saavedra M.D., Egea J., León R., Villarroya M. (2011). *N*-Acylamino-phenothiazines: Neuroprotective agents displaying multifunctional activities for a potential treatment of Alzheimer’s disease. Eur. J. Med. Chem..

[20] González-Muñoz G.C., Arce M.P., López B., Pérez C., Villarroya M., López M.G., García A.G., Conde S., Rodríguez-Franco M.I. (2010). Old phenothiazine and dibenzothiadiazepine derivatives for tomorrow’s neuroprotective therapies against neurodegenerative diseases. Eur. J. Med. Chem..

[21] Pohjala L., Utt A., Varjak M., Lulla A., Merits A., Ahola T., Tammela P. (2011). Inhibitors of alphavirus entry and replication identified with a stable Chikungunya replicon cell line and virus-based assays. PLoS ONE.

[22] Kaur P., Chu J.J.H. (2013). Chikungunya virus: An update on antiviral development and challenges. Drug Discov. Today.

[23] Pluta K., Jeleń M., Morak-Młodawska B., Zimecki M., Artym J., Kocięba M. (2010). Anticancer activity of newly synthesized azaphenothiazines in NCI’s anticancer screening. Pharmacol. Rep..

[24] Morak-Młodawska B., Pluta K., Matralis A.N., Kourounakis A.P. (2010). Antioxidant activity of newly synthesized 2,7-diazaphenothiazines. Arch. Pharm. Chem. Life Sci..

[25] Zimecki M., Artym J., Kocięba M., Pluta K., Morak-Młodawska B., Jeleń M. (2009). Immunosupressive activities of newly synthesized azaphenothiazines in human and mouse models. Cell Mol. Biol. Lett..

[26] Morak-Młodawska B., Pluta K., Zimecki M., Jeleń M., Artym J., Kocięba M. (2015). Synthesis and selected immunological properties of 10-substituted 1,8-diazaphenothiazines. Med. Chem. Res..

[27] Morak-Młodawska B., Pluta K., Jeleń M. (2015). Estimation of the lipophilicity of new anticancer and immunosuppressive 1,8-diazaphenothiazine derivatives. J. Chromatogr. Sci..

[28] Morak-Młodawska B., Pluta K., Latocha M., Jeleń M. (2016). Synthesis, spectroscopic characterization, and anticancer activity of new 10-substituted 1,6-diazaphenothiazines. Med. Chem. Res..

[29] Artym J., Kochanowska E., Kocięba M., Zaczyńska E., Zimecki M., Jeleń M., Morak-Młodawska B., Pluta K. (2016). Selected azaphenothiazines inhibit delayd type hiper-sensivity and carrageenan reaction in mice. Int. Immunopharmacol..

[30] Roman G. (2015). Mannich bases in medicinal chemistry and drug design. Eur. J. Med. Chem..

[31] Bisi A., Meli M., Gobbi S., Rampa A., Tolomeo M., Dusonchet L. (2008). Multidrug resistance reverting activity and antitumor profile of new phenothiazine derivatives. Bioorg. Med. Chem..

[32] Morak-Młodawska B., Pluta K., Latocha M., Jeleń M., Kuśmierz D. (2016). Synthesis and anticancer and lipophilic properties of 10-dialkylaminobutynyl derivatives of 1,8- and 2,7-diazaphenothiazines. J. Enzyme Inhib. Med. Chem..

[33] Morak-Młodawska B., Pluta K., Latocha M., Suwińska K., Jeleń M., Kuśmierz D. (2016). 3,6-Diazaphenothiazines as potential lead molecules—Synthesis, characterization and anticancer activity. J. Enzyme Inhib. Med. Chem..

[34] Zhang J., Chen M., Wenzhi Z., Okechukwu N.P., Morak-Młodawska B., Pluta K., Jeleń M., Md Akim A., Ang K.-P., Ooi K.K. (2017). 10*H*-3,6-Diazaphenothiazines induce G2/M phase cell cycle arrest, caspase-dependent apoptosis and inhibits cell invasion of A2780 ovarian carcinoma cells through regulation on NF-κB and [BIRC6-XIAP] complexes. Drug Des. Develop. Ther..

[35] Hemann M.T., Lowe S.W. (2006). The p53-BCL-2 connection. Cell Death Differ..

[36] Mirzayans R., Andrais B., Scott A., Murray D. (2012). New insights into p53 signaling and cancer cell response to DNA damage: Implications for cancer therapy. J. Biomed. Biotech..

[37] Scorrano L., Korsmeyer S.J. (2003). Mechanisms of cytochrome c release by proapoptotic BCL-2 family members. Biochem. Biophys. Res. Commun..

[38] Vousden K.H., Lu X. (2002). Live or let die: The cell’s response to p53. Nat. Rev. Cancer.

[39] Zhu A.K., Zhou H., Xia J.Z., Jin H.C., Wang K., Yan J., Zuo J.B., Zhu X., Shan T. (2013). Ziyuglycoside II-induced apoptosis in human gastric carcinoma BGC-823 cells by regulating Bax/Bcl-2 expression and activating caspase-3 pathway. Braz. J. Med. Biol. Res..

