# Supplementary material for: Synthesis, Anticancer Activity, and Apoptosis Induction of Novel 3,6-Diazaphenothiazines
Source: Molecules. 2019 Jan 12;24(2):267. doi: 10.3390/molecules24020267 (PMC6359424; doi:10.3390/molecules24020267)
Supplement: Supplementary file 1 [file molecules-24-00267-s001.pdf]

Supplementary Material

SYNTHESIS, ANTICANCER ACTIVITY AND APOPTOSIS INDUCTION OF NOVEL 3,6-DIAZAPHENOTHIAZINES

Beata Morak-Młodawska[1]*, Krystian Pluta[1], Małgorzata Latocha[2], Małgorzata Jeleń[1], Dariusz Kuśmierz[2]

[1]The Medical University of Silesia, School of Pharmacy with the Division of Laboratory Medicine, Department of Organic Chemistry, Jagiellońska 4, 41-200 Sosnowiec, Poland,

[2]The Medical University of Silesia, School of Pharmacy with the Division of Laboratory Medicine, Department of Cell Biology, Jedności 8, 41-200 Sosnowiec, Poland.

Content 1

1. ^{1}H NMR of the most active compound 10-[4-(N,N-diethyl)amino-but-2-ynyl]-3,6-diazaphenothiazine (**4**) 2
2. ^{1}H NMR of the compound (**4**) – aliphatic part 3
3. ^{1}H NMR of the compound (**4**) – aromatic part 4
4. ^{13}C NMR of the compound (**4**) 5
5. ^{13}C NMR of the compound (**4**) – aromatic part 6
6. ^{13}C NMR of the compound (**4**) – aliphatic part 7
7. FAB MS of the compound (**4**) 8
8. HR MS of the compound (**4**) 9

1. ^{1}H NMR of the most active compound 10-[4-(N,N-diethyl)amino-but-2-ynyl]-3,6-diazaphenothiazine (**4**)

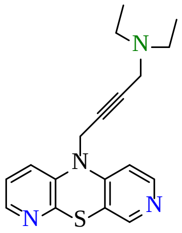

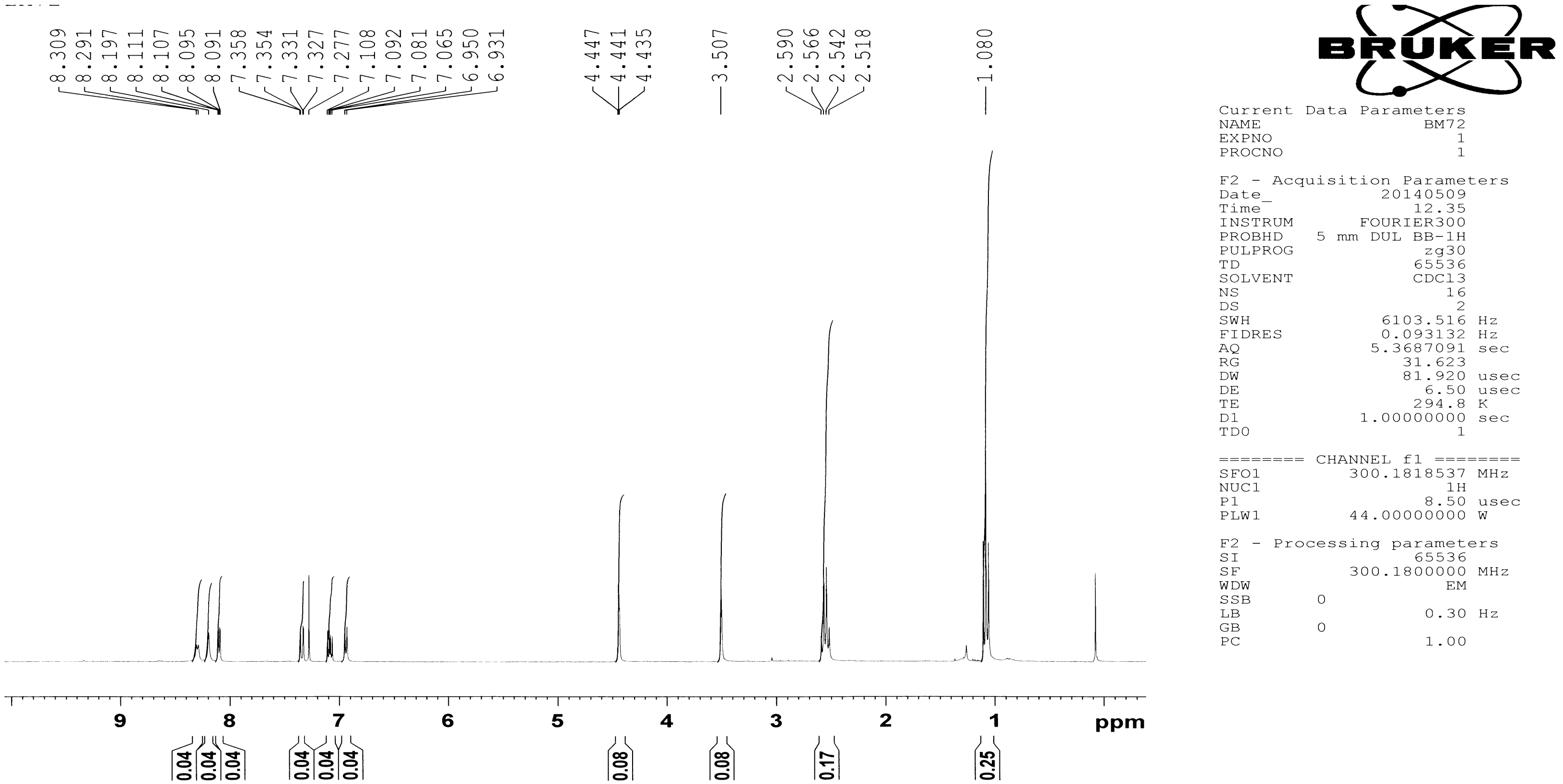

2. ^{1}H NMR of the compound (**4**) – aliphatic part

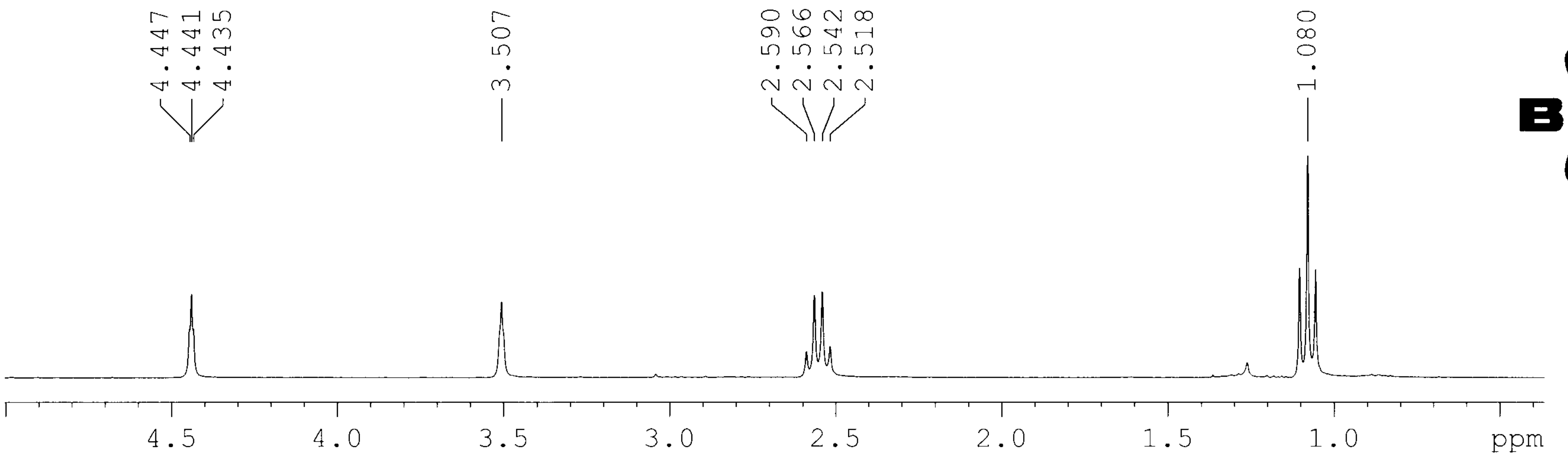

^{1}H NMR of the compound (**4**) – aromatic part

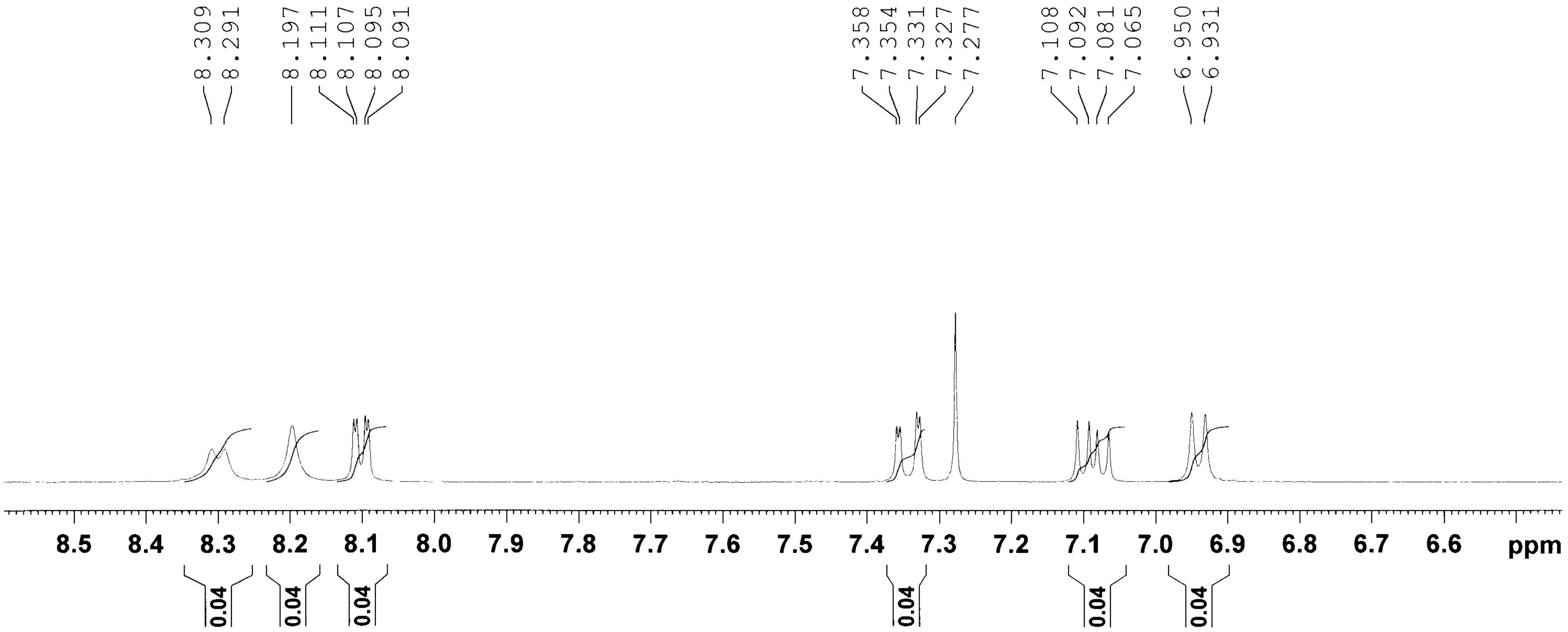

^{13}C NMR of the compound (**4**)

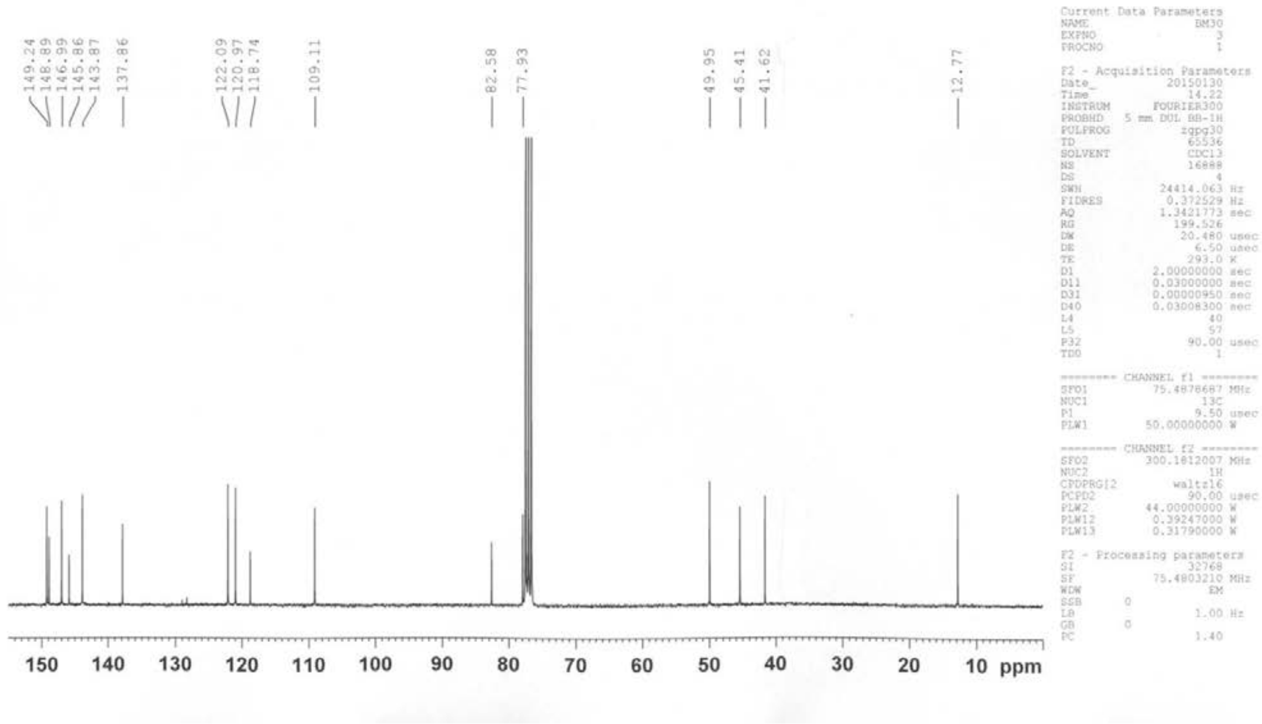

^{13}C NMR of the compound (**4**) – aromatic part

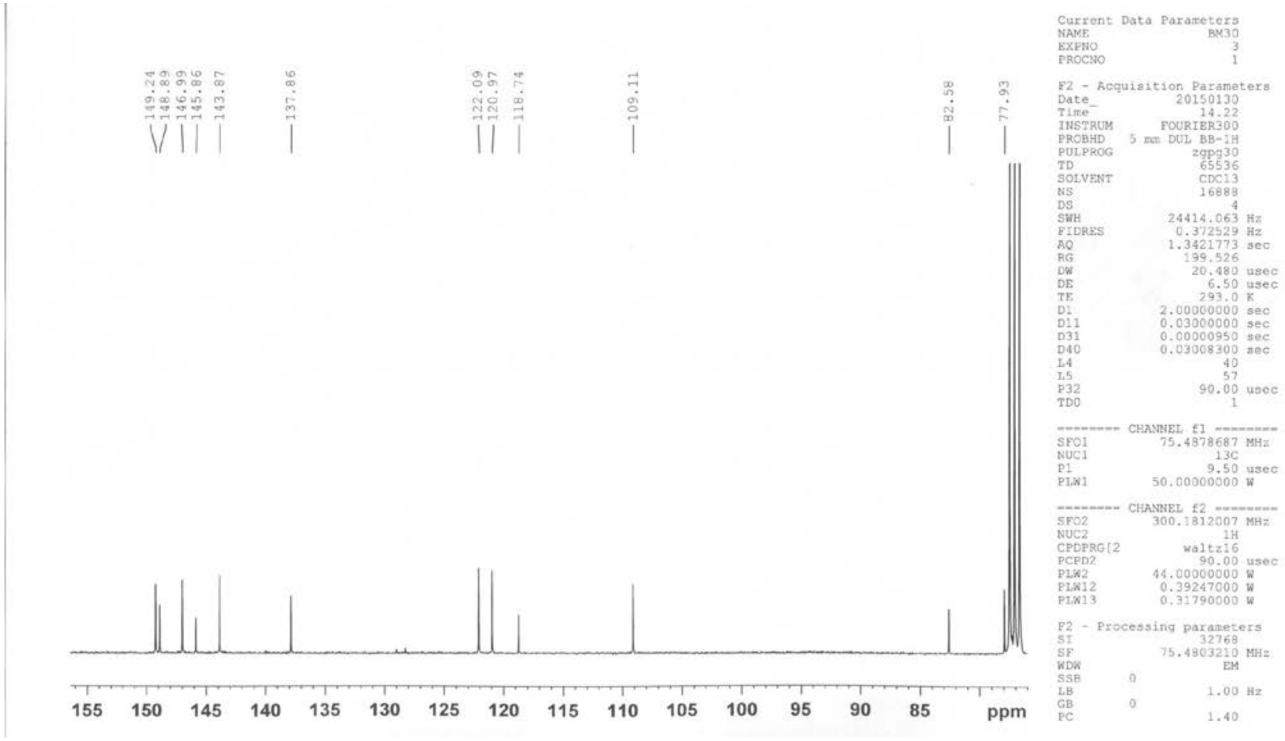

^{13}C NMR of the compound (**4**) – aliphatic part

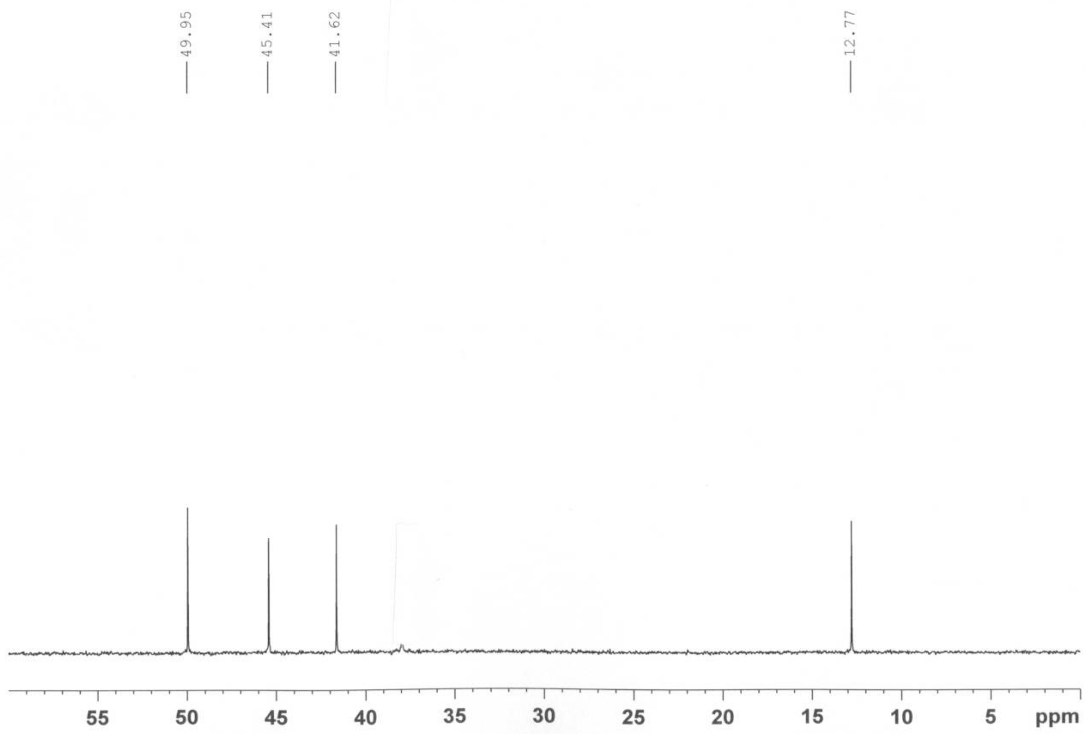

3. FAB MS of the compound (**4**)

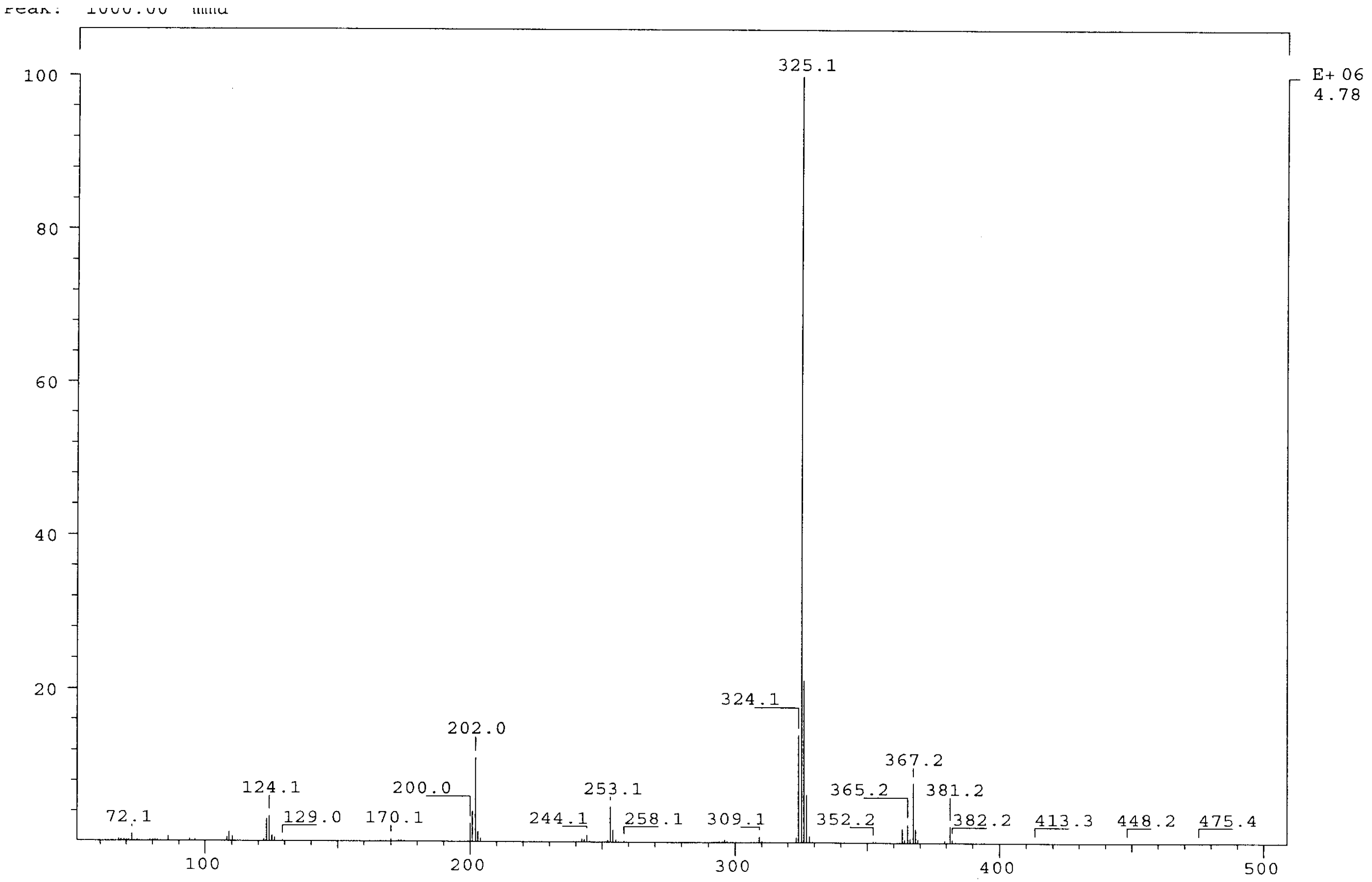

4. HR MS of the compound (**4**)

Mass Spectrum List Report

Analysis Info

Analysis Name	D:\Data\PM_20171204\BA2.d	Acquisition Date	3/21/2018 9:11:51 AM
Method	low_mass.m	Operator	KM
Sample Name	PM_20171204_tune_high_pos_before	Instrument	impact II 1825265.10082
Comment			

Acquisition Parameter

Source Type	ESI	Ion Polarity	Positive	Set Nebulizer	0.3 Bar
Focus	Active	Set Capillary	4000 V	Set Dry Heater	240 °C
Scan Begin	100 m/z	Set End Plate Offset	-500 V	Set Dry Gas	4.0 l/min
Scan End	1000 m/z	Set Charging Voltage	2000 V	Set Divert Valve	Source
		Set Corona	0 nA	Set APCI Heater	0 °C

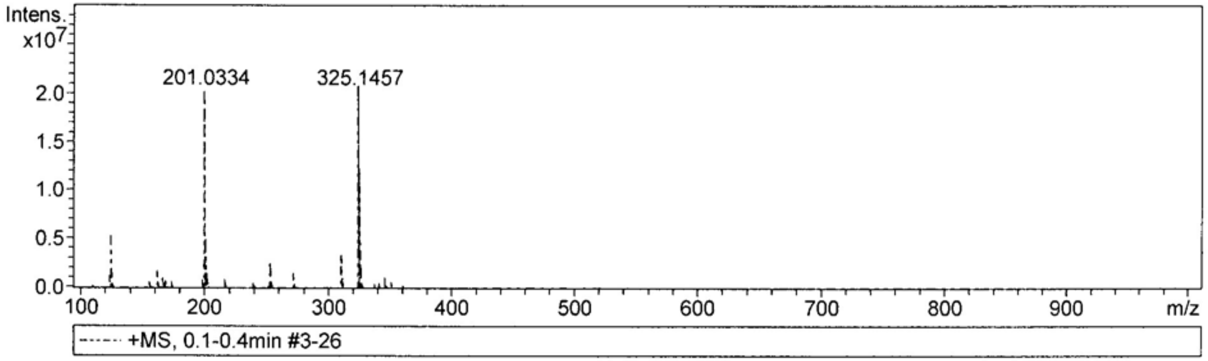

#	m/z	Res.	S/N	I	I %	FWHM
1	125.1182	26297	13316.1	4857156	23.8	0.0048
2	163.0758	28609	3520.9	1400232	6.9	0.0057
3	175.0298	26518	866.6	468366	2.3	0.0066
4	201.0334	15895	31412.9	20377586	100.0	0.0126
5	203.0293	31294	2805.6	1809982	8.9	0.0065
6	254.0718	35952	4045.7	2697730	13.2	0.0071
7	273.0775	34220	1820.4	1096647	5.4	0.0080
8	311.1294	39115	3340.8	2909928	14.3	0.0080
9	325.1457	14971	22541.4	20377596	100.0	0.0217
